# Offloading effects of a removable cast walker with and without modification for diabetes-related foot ulceration: a plantar pressure study

**DOI:** 10.1186/s13047-023-00625-z

**Published:** 2023-05-11

**Authors:** Rebekah V. Withers, Byron M. Perrin, Karl B. Landorf, Anita Raspovic

**Affiliations:** 1 Discipline of Podiatry, School of Allied Health, Human Services and Sport, La Trobe University, Melbourne, 3086 Australia; 2 La Trobe Rural Health School, La Trobe University – Bendigo Campus, Flora Hill, 3552 Australia

**Keywords:** Diabetic foot, Foot ulcer, Pressure, Plantar pressure, Kinetics, Offloading, Orthotic devices, Felt padding, Removable cast walker

## Abstract

**Background:**

Removable cast walkers (RCWs), with or without modifications, are used to offload diabetes-related foot ulcers (DRFUs), however there is limited data relating to their offloading effects. This study aimed to quantify plantar pressure reductions with an RCW with and without modification for DRFUs.

**Methods:**

This within-participant, repeated measures study included 16 participants with plantar neuropathic DRFUs. Walking peak plantar pressures at DRFU sites were measured for four conditions: post-operative boot (control condition), RCW alone, RCW with 20 mm of felt adhered to an orthosis, and RCW with 20 mm of felt adhered to the foot.

**Results:**

Compared to the control condition, the greatest amount of peak plantar pressure reduction occurred with the RCW with felt adhered to the foot (83.1% reduction, *p* < .001). The RCW with felt adhered to the foot also offered greater peak plantar pressure reduction than the RCW alone (51.3%, *p* = .021) and the RCW with felt adhered to an orthosis (31.4%, *p* = .009).

**Conclusion:**

The largest offloading effect recorded was with the RCW with felt adhered to the foot. High-quality randomised trials are now needed to evaluate the effectiveness of this device for healing DRFUs.

## Introduction

It has been estimated that 19–34% of people with diabetes mellitus will develop a diabetes related foot ulcer (DRFU) in their lifetime [1]. DRFUs are associated with a range of negative outcomes, including infection, amputation [1, 2] and poorer quality of life [3]. In 2017, global expenditure on diabetes care reached 727 billion US dollars [4], and it has been estimated that as much as a third of this relates to foot ulceration [5]. Effective management to facilitate rapid healing of DRFUs is therefore vital.

Approximately 50% of DRFUs occur on the plantar surface of the foot, with distal symmetrical polyneuropathy in the presence of repetitive mechanical pressure being key factors [6, 7]. Currently, there are no effective treatments to reverse loss of protective sensation from diabetes-related peripheral neuropathy, so reduction of damaging plantar pressure, commonly referred to as ‘offloading’, is fundamental for neuropathic plantar DRFUs [8–10].

To achieve offloading, a variety of devices are used, which offer a range of pressure reduction [11, 12]. Non-removable devices such as Total Contact Casts (TCCs) and instant TCCs (iTCCs) provide large amounts of pressure offloading and are most effective at healing DRFUs [9–11, 13]. Accordingly, non-removable devices are considered the gold-standard for treating neuropathic plantar DRFUs and are recommended in evidence-based clinical guidelines as the first-line offloading intervention [10, 14–17]. However, one study found that less than 2% of high-risk foot services in the USA utilise TCCs in DRFU management [18] and another study found that these devices are not commonly used in Australian high-risk foot settings [12].

Reasons for this underutilisation include the perceived risk of new ulceration secondary to TCC use, inability to visualise the ulcer whilst the TCC is in place, and availability of clinicians trained in the application of TCCs [12, 14, 18, 19]. In addition, there are patient-related factors that impact offloading selection, which include limitations to mobility that may, for example, make a TCC an unacceptable falls risk or cause problems driving a vehicle [12]. Therefore, whilst non-removable devices achieve the best offloading and healing outcomes, there may be significant limitations for their use in some patients and some settings. Accordingly, removable devices, such as postoperative footwear, felt padding and removable cast walkers (RCWs) are used because they bypass some of these barriers [12, 18]. Nevertheless, the pressure offloading and healing effects of these alternative devices has not been extensively researched [10, 14, 15]. While some data exists relating to the offloading effects of postoperative footwear, felt padding [20], and RCWs and RCWs made irremovable [13, 21], there is limited rigorous data investigating RCWs used in combination with adhesive felt padding or orthotic devices, which are commonly used additions or modifications to RCWs.

Therefore, the aim of this study was to investigate the effects on plantar pressure of an RCW with and without modification for plantar, neuropathic DRFUs.

## Methods

### Participants

The study used a within-participant, repeated measures design with data collected across multiple sites of Northern Health in Melbourne, Australia. Ethical approval was granted by Austin Health’s Human Research Ethics Committee (HREC/17/Austin/189) with subsequent approval from the College of Science, Health and Engineering Human Ethics Sub-Committee at La Trobe University. Written informed consent was obtained from all participants prior to beginning data collection. A sample size of 16 participants was pre-specified, which provided an 80% likelihood of detecting a clinically important difference between interventions of 100 kPa in peak plantar pressure (standard deviation of 100 kPa and alpha level set at 0.05) [22].

Recruitment took place via direct approach to patients from acute and subacute high-risk foot services within Northern Health. This included a search of electronic patient files, with a preliminary screen to meet selection criteria. A face-to-face or phone contact was then completed with the patient to discuss involvement in the study. Participants were included if they were over 18 years of age, capable of providing informed consent, had a diagnosis of diabetes mellitus, and had an active, plantar neuropathic foot ulcer for at least four weeks.

Wounds were assessed based on the International Working Group on the Diabetic Foot (IWGDF) PEDIS Ulceration Classification criteria [23] and were included if they met: Perfusion Grade 1–2; Depth Grade 1–2; Infection Grade 1; and Sensation Grade 2 (Table 1).

**Table 1:** Ulcer inclusion criteria based on the IWGDF PEDIS diabetes foot ulceration classification criteria [23]

Grade of each criteria	Description of the grade
**Perfusion Grade 1–2**	No symptoms of peripheral arterial disease in combination with: • Palpable dorsalis pedis and posterior tibial artery pulses or • Ankle-brachial index from 0.9 to 1.10 or • Toe-brachial index > 0.6 or • Transcutaneous oxygen pressure (tcpO_2_) > 60 mm Hg Symptoms or signs of PAD, but not of critical lime ischaemia (CLI): • Presence of intermittent claudication OR • Ankle-brachial index < 0.9 but with ankle pressure > 50 mm Hg • Toe-brachial index < 0.6 but systolic toe blood pressure > 30 mm Hg
**Depth Grade 1–2**	• Superficial full-thickness ulcer, not penetrating any structure deeper than the dermis or • Deep ulcer, penetrating below the dermis to subcutaneous structures, involving fascia, muscle or tendon
**Infection Grade 1**	• No symptoms or signs of infection
**Sensation Grade 2**	• Absent pressure sensation, determined with a 10-g monofilament, on two out of three sites on the foot’s plantar surface as described in the International Consensus on the Diabetic Foot or • Absent vibration sensation on the hallux using a 128-Hz tuning fork or vibration threshold > 25 V

Participants were deemed ineligible if they: were unable to walk 10 m without the use of a walking aid, had critical limb ischemia as defined by a toe pressure less than 30 mm Hg, an ulcer probing to bone, or infection as marked by the cardinal signs of redness, heat, swelling, pain and purulent exudate [24]. Individuals who did not speak and understand English and who were unable to communicate without an interpreter were excluded to ensure informed consent and adequate communication during the study.

Participant baseline characteristics including age, sex, diabetes duration and type, and amputation history were recorded from electronic records. Amputation history was included as it has been shown that patients with a history of partial foot amputations have high plantar pressures than those without amputations due to biomechanical compensation [25]. DRFU dimensions were recorded on the day of data collection with a sterile ruler (Puritan DM Stick®, Guilford, ME). Body mass index (BMI) was calculated on the day of data collection using an electronic calculator (National Heart Foundation of Australia^©^ BMI calculator), which divides body weight in kilograms by height in metres squared. In the event baseline characteristic details were not available in the participant’s electronic health records, they were confirmed with the participant or local general practitioner.

### Offloading conditions

**Fig. 1:**
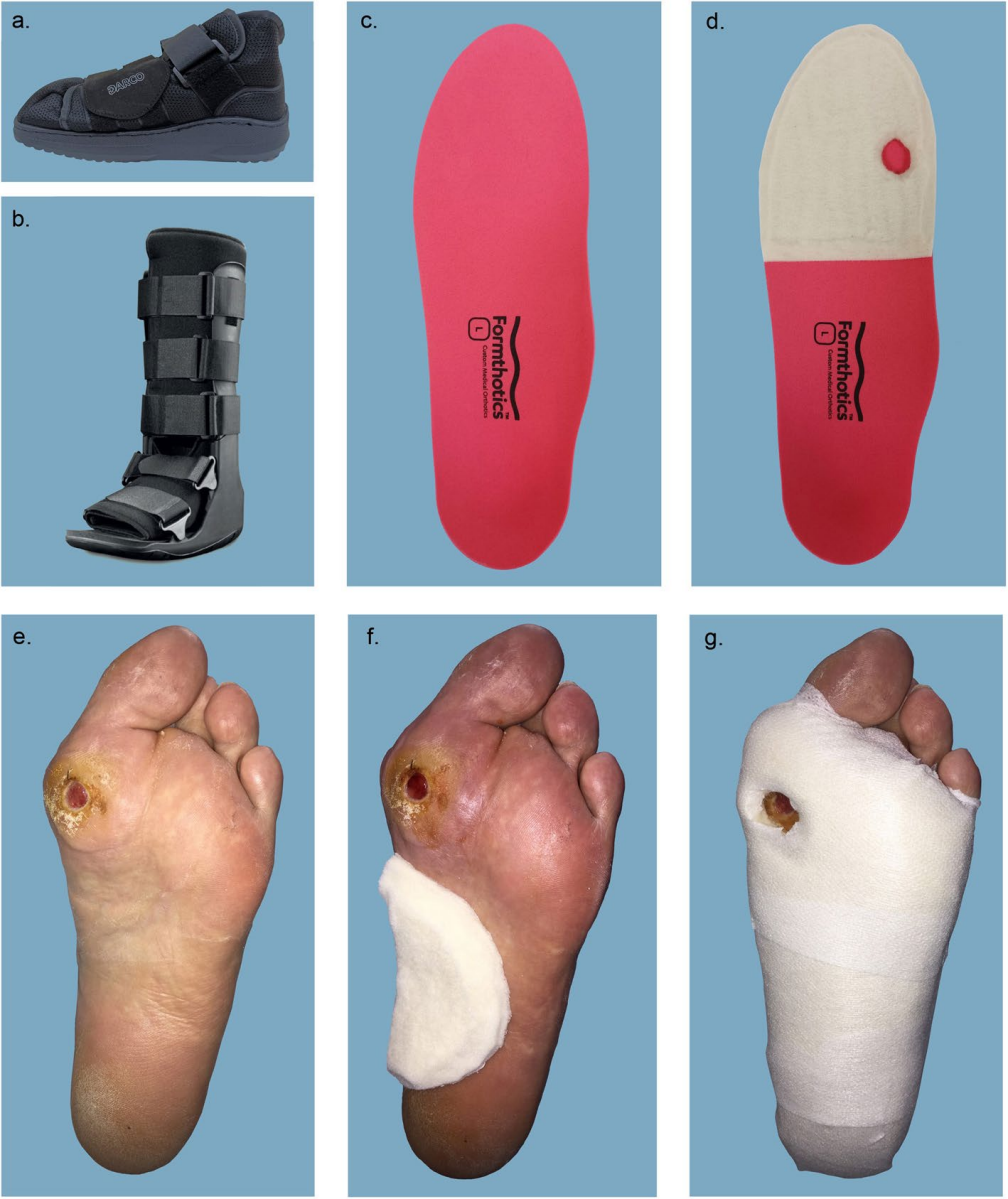
Conditions assessed in the study: **a** Postoperative footwear with the lining removed (the control), **b** RCW alone, **c** and **d** RCW with 20 mm of felt padding adhered to an orthosis (RCW with felt to orthosis), **e–g** RCW with 20 mm of felt padding adhered to the foot (RCW with felt to foot)

All participants were measured in four conditions, one of which was a control. Selection of these conditions was based on previous research [12]. The conditions included the following (Fig. 1):

(i) Postoperative footwear with the lining removed (the control),
(ii) RCW alone,
(iii) RCW with 20 mm of felt padding adhered to an orthosis (RCW with felt to orthosis),
(iv) RCW with 20 mm of felt padding adhered to the foot (RCW with felt to foot).

For the control condition, participants were measured in a post-operative boot (APB™ All Purpose Boot, DARCO, Huntington, WV) on the limb with ulceration present. This footwear was selected as its design accommodates varying degrees of anatomical deformity, and with the lining removed, it was considered to have limited influence on plantar pressures. For the remaining offloading conditions a RCW was used (ProCare® XcelTrax Standard Tall Walking Brace, DJO Global, Vista, CA). The RCW was only worn on the limb with active ulceration; all participants wore their regular footwear on the contralateral limb for all conditions.

The orthosis used in the study was a prefabricated full length, firm (180 kg/m^3^) closed cell polyethylene foam device (Foot Science International, Christchurch, New Zealand), which contours the plantar surface of the foot, including the arch and heel. While the insoles can be heat-moulded, they were not for this study. When used in combination with the RCW, the orthosis was placed inside the liner of the RCW.

The felt padding used for the study was made of semi-compressed felt with an adhesive backing (Aetna Felt Corporation, Allentown, Pennsylvania, USA). All felt padding was 20 mm thick with an aperture at the ulcer site where appropriate. The borders of the aperture site under the ulcer were as close as possible to the margins of the ulcer. The edges of the felt were bevelled to avoid unintentional high pressure areas at the margin of the aperture. For the RCW with felt adhered to an orthosis condition, the felt pad dimensions depended on where the DRFU was located. For a hallux or forefoot ulcer, the proximal border of the pad was 1–2 cm distal to the metatarsal bases and the distal border was the distal margin of the orthosis. For a midfoot or rearfoot ulcer, the proximal border of the pad was the anterior margin of the heel and the distal border was the distal margin of the orthosis (an aperture was used for midfoot ulcers, but not for rearfoot ulcers as there was no felt directly under the heel). The orthosis with the felt pad applied was positioned inside the RCW before it was applied to the limb. For the RCW with felt adhered to the foot condition, the proximal border of the pad was the anterior margin of the heel and the distal border was the webspaces of the toes. When necessary, the pad was secured to the foot with hypoallergenic tape (Hypafix, Smith & Nephew, Andover, MA) to ensure the felt did not shift during data collection. The RCW was applied to the limb after the felt pad was adhered to the foot. Foot ulcers were covered with a thin gauze, sterile protective dressing.

### Randomisation and blinding

To minimise ordering effects, the order that the offloading conditions were measured was randomised. Participants and investigators were not blinded to the offloading conditions, however the pressure measurements obtained with the pedar-X® were objective, reducing the risk of bias.

### Plantar pressure measurement equipment

Plantar pressure data were collected with an in-shoe measurement system, the pedar-X® (Novel, Munich, Germany), which has been found to be valid and reliable [26–29]. The pedar-X® insoles are 2 mm thick, constructed with 99 capacitive sensors arranged in a grid formation and record peak plantar pressures in kilopascal (kPa) units. The pedar-X® insoles were calibrated using the trublu® calibration system as per manufacturer guidelines (Novel, Munich, Germany). The sampling frequency of the system was 50 Hertz. All plantar pressure measures were obtained in accordance with manufacturer guidelines (Novel, Munich, Germany).

### Measurement procedure

Insoles of the pedar-X® system were placed in each offloading intervention as close to the foot as the intervention would allow to standardise the positioning of the pedar-X® insoles and obtain the most accurate plantar pressure measurement. However, due to the nature of the 20 mm felt padding adhered to the foot condition, the pedar-X® insoles were unable to be placed directly against the foot. Instead, they were placed between the felt and RCW, which was based on previously published protocols [20, 22]. Participants were granted five minutes to walk and acclimatise to the device with the pedar-X® in place prior to beginning data collection. Before the initial trial for each condition, pedar-X® insoles were zeroed as per manufacturer guidelines (Novel, Munich, Germany). Participants were then measured whilst walking at a comfortable pace along a pre-measured, flat 10 m walkway. To control for variation in walking speed, each trial was timed and if not within ± 5% of the first trial recorded it was not included, and the participant was required to complete the trial again. To limit the effects of acceleration and deceleration, only the middle three steps of each trial were analysed.

### Outcome measures

The primary outcome measure was peak plantar pressure (kPa), which was assessed at the active DRFU site of each participant. This outcome was selected as peak plantar pressure has been shown to be predictive of tissue trauma, ulcer formation, delayed healing and ulcer recurrence [30, 31]. Force–time integral and pressure–time integral were not analysed as they have high interdependency with peak pressure [32, 33].

The secondary outcome measures of contact area (cm^2^) and contact time (ms^−1^) were also included. Contact area provided data on whether plantar pressure changes were linked to change in the plantar contact area with the offloading devices tested [20]. Contact time was measured to cross-check consistency of walking speed during trials [20].

### Data processing and analysis

Raw plantar pressure data obtained with the pedar-X® system was processed using the Novel multi-mask package (Novel, Munich, Germany). This enabled a single mask to be placed over a specific region of interest for each participant (i.e. the DRFU site). This mask subsequently provided an area of comparison for the four offloading conditions. The secondary outcomes measures of contact area and contact time were processed using a whole foot mask. All statistical analyses were undertaken using the Statistical Package for the Social Sciences (SPSS) Version 23 (IBM Corp, Somers, NY, USA).

Normality of data was confirmed by assessing histograms, skewness and kurtosis values, and Shapiro-Wilks statistical tests [34]. Four outliers were identified, all in peak plantar pressure. Outliers were addressed by replacement with the next non-outlier value. Between-group differences for peak pressure were expressed as mean differences with 95% confidence intervals, and to provide easier comparison, as percentage differences. One-way repeated-measures ANOVAs were used to explore the effects of all offloading conditions on peak plantar pressures (ulcer), contact area (whole foot) and contact time (whole foot). Mauchly’s test was assessed to address the assumption of sphericity, and where it was violated, the Greenhouse–Geisser correction was used for the *F*-statistic, degrees of freedom and the *p*-value. Significant ANOVA findings were explored using Bonferroni-adjusted pairwise comparisons. The threshold for statistical significance was set at 0.05.

## Results

### Participant characteristics

A total of 16 participants with 16 ulcers were recruited to the study. Participant and ulcer characteristics are outlined in Table 2. Thirteen ulcers were located under the forefoot, two were under the rearfoot, one was under the midfoot and one was under the hallux.

**Table 2:** Participant characteristics (*N* = 16)

Characteristic	Values
Age in years: mean (± SD)	59.6 (± 12.5)
Sex: n (%)	
Male	13 (81.3%)
Female	3 (18.7%)
Weight in kg: mean (± SD)	101.3 (± 28.0)
Height in cm: mean (± SD)	177.7 (± 10.2)
Body Mass Index in kg/m^2^: mean (± SD)	31.2 (± 5.1)
Diabetes duration in years: mean (± SD)	16.9 (± 9.2)
Diabetes Mellitus type: n (%)	
Type 1	3 (18.7%)
Type 2	13 (81.3%)
Previous amputation: n (%)	
None	10 (62.5%)
Digit	5 (31.25%)
Ray	1 (6.25%)
Ulcer location: n (%)	
Hallux	1 (6.25%)
Forefoot	12 (75%)
Midfoot	1 (6.25%)
Rearfoot	2 (12.5%)
Ulcer dimensions in mm: mean (± SD)	
Length	15.2 (± 12.1)
Width	10.2 (± 6.4)
Depth	2.5 (± 2.7)

### Walking speed

There was no significant difference in contact time between the conditions (F_1.6, 23.4_ = 0.940, *p* = 0.384), so walking speed was consistent (Table 3). Therefore, any plantar pressure differences that were found can be directly attributed to the test condition, not due to changes in contact time.

**Table 3:** Means and standard deviations for peak plantar pressure, contact area and contact time (*N* = 16)

	**Peak pressure (kPa) (ulcer site)**		**Contact area (cm**^**2**^**) (whole foot)**		**Contact time (ms**^**−1**^**) (whole foot)**	
	Mean	SD	Mean	SD	Mean	SD
**Control**	386.6	148.8	144.2	29.0	780.5	88.3
**RCW**	133.9	92.8	139.3	40.5	787.9	92.6
**RCW with felt to orthosis**	95.0	33.5	150.3	40.5	791.6	115.8
**RCW with felt to foot**	65.2	35.9	148.2	37.9	805.8	142.7

### Differences in peak pressure and contact area between the conditions

Overall, there was a significant difference in peak plantar pressure at the ulcer site (F_1.6, 23.3_ = 58.202, *p* < 0.001) between the four conditions (Table 3). There was no significant difference in contact area (F _3, 13_ = 5.33, *p* = 0.100) between the conditions.

**Fig. 2:**
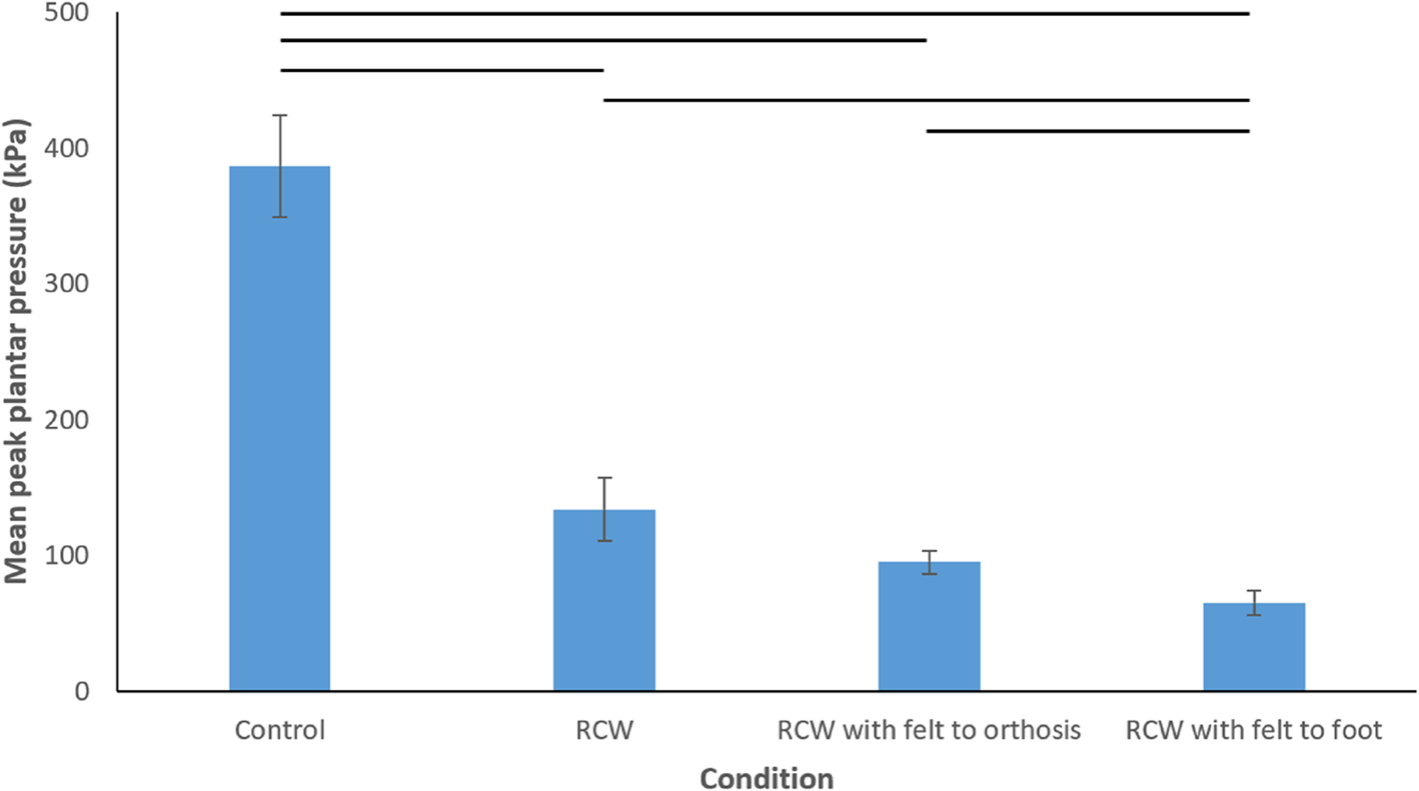
Comparison of mean peak plantar pressures for the control and three removable cast walkers (RCWs) – error bars are standard errors and horizontal lines at top of graph indicate statistically significant differences between two conditions

Pairwise comparisons revealed that peak plantar pressures were significantly lower in all RCW conditions compared to the control condition (Table 4 and Fig. 2). The RCW with felt adhered to the foot was found to provide a significantly greater reduction in peak plantar pressure compared to all other conditions measured. This was most notable when the RCW with felt adhered to the foot was compared to the control condition, where 83.1% less peak pressure was observed. When the three RCW conditions were compared, there was significantly less peak pressure (51.3% less) with the RCW with felt adhered to the foot compared to the RCW alone and the RCW with felt adhered to an orthosis (31.4% less). No significant difference in peak pressure was observed when the RCW with felt adhered to an orthosis was compared to the RCW alone.

**Table 4:** Comparisons between conditions for peak pressure (*N* = 16)

Comparison	Mean difference in peak pressure (kPa)^a^	95% CI	*P*-value	% difference between conditions^b^
**Control – RCW**	252.7	150.5 to 354.8	< .001	65.4% decrease
**Control – RCW with felt to orthosis**	291.6	189.4 to 393.7	< .001	75.4% decrease
**Control – RCW with felt to foot**	321.4	208.4 to 434.4	< .001	83.1% decrease
**RCW – RCW with felt to orthosis**	38.9	-17.9 to 95.7	.331	29.1% decrease
**RCW – RCW with felt to foot**	68.8	8.4 to 129.1	.021	51.3% decrease
**RCW with felt to orthosis – RCW with felt to foot**	29.8	6.5 to 53.2	.009	31.4% decrease

^a^Mean difference equates to the peak pressure of the first condition listed minus the peak pressure of the second condition listed

^b^A decrease in percentage indicates that the latter condition recorded a lower mean than the former condition

## Discussion

Findings for this study show that compared to the control condition, the RCW with and without modification can produce large reductions in peak pressure at DRFU sites. There was a 65.4% reduction with an RCW alone, a 75.4% reduction with an RCW with felt adhered to an orthosis, and an 83.1% reduction in peak pressure for the RCW with felt adhered to the foot. For the RCW with felt adhered to the foot condition, the offloading found in this study is only slightly less than offloading found for TCCs and iTCCs when compared to control conditions in other studies [11, 13].

When comparing the RCW conditions, the RCW with felt adhered to the foot led to significantly less plantar pressure compared to the RCW alone and RCW with felt adhered to an orthosis (51% and 31%, respectively). In addition, the ease of application and degree of customisation offered with the felt adhered to foot modification may render it useful where RCWs are being considered in DRFU management and there is the time and necessary materials and equipment to do so.

The removable nature of an RCW has the added benefit of overcoming perceived barriers to irremovable offloading devices such as the inability to assess DRFUs regularly or the risk of iatrogenic DRFUs. From a patient perspective, removable devices may be preferable as they do not have as profound an impact on lifestyle and capacity to carry out activities of daily living. However, because they are easily removed, there is the possibility that patients will not wear them as frequently (i.e. non-compliance), which can result in suboptimal healing [11, 35, 36]. This is pertinent to optimal DRFU management as patient engagement with self-care [37, 38], including wearing offloading devices is crucial [38–40].

Although this study found that RCWs with modification lead to substantially less plantar pressure under DRFUs, high quality randomised trials that evaluate their effectiveness are now required. Such trials should also monitor compliance. In addition, adverse events should be closely monitored to ensure they can be used safely. Without such evaluations it cannot yet be concluded that they are effective at healing DRFUs or safe.

A key strength of this study is the interventions investigated were selected pragmatically to reflect devices and modifications commonly used in practice. In addition, they do not require additional credentialing or training to apply. Other strengths of this study include the order of application was randomised and the pedar-X® system produces objective data, which reduces the likelihood of bias from the unavoidable lack of blinding.

However, there are also three important limitations. Firstly, the conditions were not measured after a prolonged period of wear, so we cannot rule out less plantar pressure reduction over time, particularly with compression of the felt. Secondly, the pedar-X® system is only able to measure vertical pressure [26–29], and ulcerogenic forces are likely more complicated, including components such as shear forces [41]. However, it should be noted that technology to measure other ulcerogenic forces does not yet exist. Furthermore, issues with spatial resolution of plantar pressure system have been raised before [42–44]. Nonetheless, in-shoe pressure measurement systems are deemed the best available option for measuring forces acting between offloading devices and the foot [45, 46] and have been frequently used in the last 20 years to do so [13, 17, 20]. Thirdly, we acknowledge that the placement of the pedar-X insoles between the different offloading conditions assessed may have introduced some error into the plantar pressure output. For example, for the RCW with felt to orthosis condition, the pedar-X insole had to be positioned on top of the felt, which was a different position to the other conditions where the pedar-X insole was positioned on top of the flat insole of the shoe/RCW (for the control and RCW alone conditions) or the orthosis (for the RCW with felt to foot condition). This may have led to some resistance by the pedar-X insole over the aperture in the felt, which possibly could have introduced some error. However, there is currently no evidence for this phenomenon, so this is speculative. Importantly, we were unable to test the conditions any other way than the way we did, but this potential source of error should be considered when viewing our findings.

## Conclusion

This study shows that RCWs are effective at reducing plantar pressure from DRFUs. The largest plantar pressure reduction compared to the control condition was 83.1%, which was achieved with an RCW with 20 mm of felt adhered to the foot (with an aperture at the DRFU site). While the offloading effects of this device is slightly less than recommended first-line interventions of TCCs and iTCCs, its practicality renders it useful when non-removable devices are contraindicated or unavailable, or if RCWs are being modified with the view to further reduce plantar pressure. Randomised trials are needed to evaluate the effectiveness and safety of RCWs with and without modifications.

## Data Availability

The datasets generated during and/or analysed during this study are available from the corresponding author on reasonable request.
